# I’m Still Standing: Body Sway, Interpersonal Distance, and Social Anxiety – A Proof of Principle

**DOI:** 10.32872/cpe.15365

**Published:** 2025-08-29

**Authors:** Wolf-Gero Lange, Muriel A. Hagenaars

**Affiliations:** 1 Behavioural Science Institute, Radboud University Nijmegen, Nijmegen, The Netherlands; 2 Department of Clinical Psychology, Faculty of Social and Behavioural Sciences, Utrecht University, Utrecht, The Netherlands; Friedrich-Alexander-Universität Erlangen-Nürnberg, Erlangen, Germany

**Keywords:** social anxiety, freezing behavior, interpersonal distance, stabilometric force platform, body sway, avoidance

## Abstract

**Background and Objectives:**

Cognitive models suggest that individuals with high degrees of social anxiety (SAs) tend to incorrectly interpret (ambiguous) social cues as negative evaluations and thus justifying their fears. It is assumed that subtle behaviors of SAs may give rise to factual negative evaluations, but it is unclear which kind of behaviors that may be. We tested whether automatic motivational behavior becomes disrupted when degree of social anxiety increases, expecting higher social anxiety to be associated with more threat-related ‘freezing’ (reduction of body sway) and backward leaning (avoidance).

**Method:**

Of 87 participants with varying degrees of social anxiety, body sway was recorded by means of a stabilometric platform, while a fe-/male experimenter was gradually approaching.

**Results:**

Higher levels of social anxiety were related to an *increase* of body sway at an interpersonal distance of 260 to 120cm. No avoidant backward-leaning occurred.

**Limitations:**

Predictability of set-up and knowledge of escape options may have undermined participants’ experience of the situation as highly socially threatening. Unease-, rather than fear-related behavior may have been the result.

**Conclusions:**

The results indicate that SAs seem to show an increase in uneasy, nervous movement when approached by strangers. Whether that provokes the negative evaluation SAs fear most, still needs to be investigated.

## Highlights

- Stranger approach paradigm was used to study freezing and avoidance behavior in social anxiety.
- Objective changes in body posture were assessed by a stabilometric force platform.
- Higher social anxiety was related to increased body sway during stranger approach.
- Freezing behavior was associated to state but not to (trait social) anxiety.

Social anxiety disorder (SAD) is a common and highly distressing disorder (e.g., Fehm et al., 2005; Kennedy et al., 2009; Perry et al., 2016) that is characterized by an excessive fear of negative evaluation by others in social or performance situations (American Psychiatric Association, 2013, 2022). Until recently, it has been predominantly conceptualized as a condition of ‘distorted or biased information processing’ (Clark & Wells, 1995; Hofmann, 2007; Rapee & Heimberg, 1997) based on the idea that individuals with high degrees of social anxiety (SAs) have a tendency to see social danger where in fact there is none. This tendency is thought to cumulate and eventually maintain SAD (Beard & Amir, 2008; Heeren et al., 2012; Schmidt et al., 2009).

Yet, there is growing evidence that SAs *are* truly evaluated in a negative way. Heerey and Kring (2007) found that interactions with SAs were characterized by, e.g., nervous fidgeting, or poor reciprocity of smiling (Heerey & Kring, 2007). As a consequence, conversations with SAs evoked increased negative affect in interaction partners (Alden & Wallace, 1995; Creed & Funder, 1998; Meleshko & Alden, 1993; Voncken & Bögels, 2008).

While the above-mentioned behaviors are more of a deliberate nature, the question remains whether SAs show disruptions in *subtle*, more automatic behaviors in social interaction. Research by Asher et al. (2020) showed that non-verbal behavioral synchrony increased in non-anxious couples during conversation while it decreased in anxious/non-anxious dyads. As behavioral synchrony is associated with increased positive affect (Tschacher et al., 2014) and increased sense of rapport (Miles et al., 2009), a lack of syncing with others may actually lead to the effect that SAs fear most.

In a different line of research, Givon-Benjio and Okon-Singer (2020) reported that increased degrees of social anxiety were related to a preference for larger interpersonal distance (IPD) *and* a tendency to underestimate interpersonal distances to strangers when compared to friends (see Givon-Benjio et al., 2020 for replication in individuals diagnosed with SAD).

While keeping more IPD *to* others may be a means to downregulate anxiety, breaches of an SA’s personal space *by* others should increase anxiety (Perry et al., 2013). Accordingly, Wieser et al. (2010) showed that high social anxiety was associated with avoiding the gaze of a virtual male agent at 1.5m IPD, while the gazes of male agents were avoided irrespective of degree of anxiety at 0.5m. SAs also showed avoidance in the form of subtle backward head-movements irrespective of IPD (Wieser et al., 2010). In addition, closer IPD and direct gaze were associated with heart rate deceleration. These last findings are particularly intriguing as decelerations have been linked to orienting and freezing responses in the past, while *accelerations* is thought to indicate fearful or phobic responding (Klorman et al., 1977; Ruiz-Padial et al., 2005; for critical discussion, see Barry & Maltzman, 1985).

It is only since this millennium, that researchers have started to investigate human freezing behavior as reflected by reduced body sway in response to threat (Azevedo et al., 2005; Hagenaars et al. (2014a); Roelofs, 2017; Volchan et al., 2017). It is proposed that a common response to imminent (unavoidable) threat is to ‘freeze’, *before* flight or fight responses are triggered (Blanchard, 2017; Bracha, 2004; Fanselow, 2022; Fanselow et al., 1987; Hagenaars et al., 2014a). Eilam (2005), however, argued that freeze and flight responses are mutually exclusive but may, nevertheless, alternate quickly, dependent on the situation. In the same line, Hagenaars et al. (2014a), suggested that when freeze/fight/flight responding had evolved for survival purposes, it must be dynamic and flexible rather than being characterized by chronological order and lack of overlap. Accordingly, Fanselow (2022) postulates that, under more, context, type and direction of approaching threat determines the ‘adaptive defense threshold’ and thus the timing, kind and magnitude of responding.

A few studies investigated freezing-like behavior in response to social threat. Roelofs et al. (2010a) and Noordewier et al. (2020) placed healthy participants on a stabilometric force platform and/or measured their heart-rates while passively viewing neutral, angry, and happy facial expressions. Viewing angry faces was associated with freezing-related behavior, e.g., a reduction in body sway. Moreover, reduced body sway was associated with increased state anxiety. In patients with SAD, Levitan et al. (2012), found reduced body sway irrespective of stimulus type (neutral objects, social threat, generic threat). Niermann et al. (2017), investigated freezing behavior after a physiological and social stress induction (Smeets et al., 2012) and found associations between diminished recovery from freezing and increased internalizing symptoms (such as, e.g., social anxiety).

In sum, there is reason to assume that SAs show distinct postural behaviors in social interactions, and in particular when their interpersonal space is breached. Yet, research assessing postural changes in human beings has seldomly looked at the impact of a trait such as social anxiety. It is particularly remarkable that all studies used static stimuli while threatening situations are typically of a dynamic nature. To our knowledge, postural balance fluctuations have not, yet, been investigated when the cause of threat is gradually approaching.

In this proof-of-principle study we therefore investigated at what distances between participant and an approaching research assistant (RA), avoidance behaviors and/or freezing would occur and which role social anxiety would play herein. We expected increasingly marked backward swaying of the participant when the RA would come closer. Such impulsive backward swaying would indicate embodied negative evaluation (Bargh & Chartrand, 1999; Niedenthal et al., 2005). In addition, we expected that freezing responses, indicated by reduced body sway, would increase with a decreasing distance between RA and participant. Finally, we expected that freezing and simultaneous avoidance would occur earlier (at a greater distances) with higher degrees of social anxiety. The stance of the RA (moving vs still) was controlled for.

## Method

### Participants

In total 87^1^1In the time that the study was conducted, preregistrations and a priori power analyses were rather uncommon, and thus not done for the current study. For power estimations, it was common to rely on the sample sizes of comparable studies (Lakens, 2022). For the present study, thematically related articles indicated sample sizes between 20 (Rinck et al., 2010) and 148 (Kaitz et al., 2004). We strove to recruit as many participants as possible within two semesters, but at least 80. Post hoc power analyses indicated that with an effect size of η^2^ = .25 (Wieser et al., 2010) and a power of α = .80 a necessary sample size of 87 was calculated with g*power (Faul et al., 2009) which coincidentally is the number of participants that we recruited.” students from Radboud University Nijmegen (67.8% female) participated in the study with a mean age of 21.75 years (*SD* = 3.17), ranging from 18 to 41 years. The whole sample consisted of 75.7% individuals from Dutch origin, 20.9% Germans and 1.1% from other countries. With 66.7%, psychology was the predominant field of studies, followed by law (10.3%) and pedagogical sciences (8%). The remaining 15% was distributed nearly even across five other fields of studies or did not indicate a direction. The experiment took about 20–25 minutes, and, after completion, participants received a candy bar or course credit.

### Questionnaires

Before the personal space task, participants completed a general screening instrument to assess sociodemographic information (e.g., age, gender, native language, education) and their height in centimeters (cm). Prior to the experiment some state measures were assessed by means of *Visual Analogue Scales* (VASs; Bond & Lader, 1974). Participants had to indicate on a 10 cm wide slider ranging from 0 (*not at all*) to 100 (*extremely*) how anxious they were at the very moment, how tense they felt and how much they would like to escape the situation. Typically, VASs have good psychometric properties (Wewers & Lowe, 1990). In the present study Cronbach’s α was .55.

After the experimental task, participants were asked to answer questions to correct for possible experimenter related variables. Participants had to indicate by means of four VASs how friendly, sympathetic, and attractive they evaluated the research assistant (RA) ranging from 0 (*not at all*) to 100 (*very much*). In addition, they were asked to evaluate the body scent of the RA, ranging from 0 (*very unpleasant*) to 100 (*very pleasant*). In the present study, Cronbach’s α for these scales was .83.

Level of social anxiety was assessed with the Dutch self-report version (van Vliet, 1999) of the *Liebowitz Social Anxiety Scale* (LSAS; Liebowitz, 1987; Oakman et al., 2003). The participants indicated for 24 social situations (e.g., “Talking to people in authority”) on a 4-point Likert-type scale, ranging from 0 (*none*) to 3 (*severe*) how anxious they would be in these situations and how much they would avoid them from 0 (*never*) to 3 (*usually*). The psychometric properties of the scale are generally considered good (dos Santos et al., 2013; Heimberg et al., 1999) but the Dutch version has not been officially evaluated, yet (Lange et al., 2024). The internal consistencies of the total score of the LSAS measured by means of Cronbach’s α was .93 in the present study.

In order to control for depressive symptomology often associated with social anxiety (e.g., Adams et al., 2016), participants completed the Dutch version of *Center for Epidemiological Studies Depression Scale* (CES-D; Ratloff, 1977). They had to indicate how often they had experienced each of the 20 listed symptoms (e.g., “I felt depressed”) in the last week on a 4-point Likert scale ranging from 0 (*almost never*) to 3 (*almost always*). CES-D has good psychometric properties (Bouma et al., 2012). In the present study, Cronbach’s α was .92.

### Apparatus

**Figure 1:**
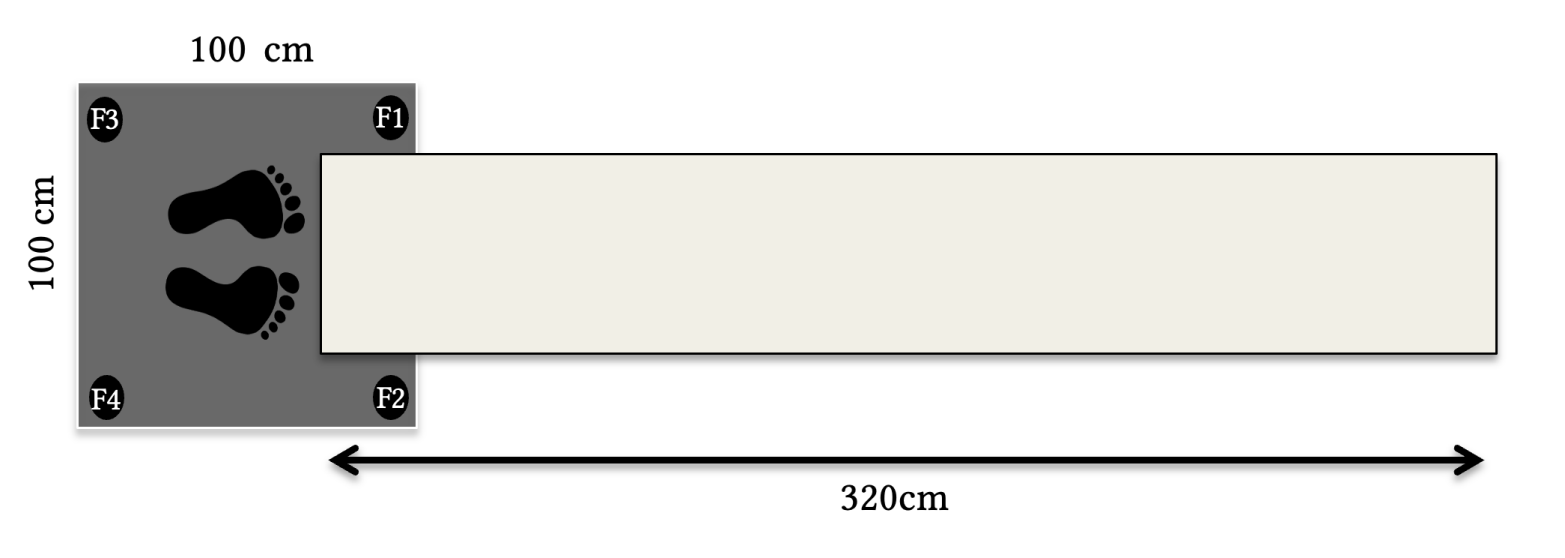
Sketch of the Balance Board Set-up and Sensor Numbering

The questionnaires were filled in online on a standard PC via the survey-platform Unipark (www.unipark.de). The stabilometric force platform (balance board [BB]) was a 1 m × 1 m custom-made strain-gauge force plate with a sampling frequency of 4 × 100 Hz, a resolution of 0.28 N/bit and a resonance frequency of 30 Hz. Changes in center-of-pressure (COP) were recorded in anterior-posterior (AP) direction as well as the medio-lateral (ML) direction by means of four pressure-sensitive sensors at each corner of the plate. As the BB was 16 cm high, a custom-made “catwalk” (3.20 m long and 50 cm wide) was used on which the RAs could approach the participants (Figure 1).

To mark the distance between participant and RA in the BB data, a bell-button with 5 m cable was connected to the laptop via a custom-made button box. All BB data were recorded by Presentation® software (www.neurobs.com). To record the participants’ distance estimations an Olympus™ digital voice recorder was used.

### Procedure

Prior to a test session, each participant was randomized to be tested by either the male or the female RA. Upon arrival participants were accompanied into the lab, were informed about the test session and were asked to give informed consent. After that, they filled in the sociodemographic information, while the RA left the room. After the RA’s return, participants were informed that they were to judge the distances between themselves and the RA who would be approaching in small steps on the catwalk. Participants were instructed to not look for spatial cues in the room but to focus on the (neutral) face of the RA. Whenever the RA had completed a step, they were asked to speak their estimation out loud but would not receive any response. The RA also explained that s/he would eventually come very close, but that the participant was to stay on the platform.

When the participants indicated that they had understood the instructions, they were asked to take off their shoes and stand loosely but straight in the middle of the BB facing towards the “cat-walk”. The RA started the software that recorded the BB signals and the voice recorder. S/He took a position on the catwalk at roughly 3.20 m from the participant (Figure 1) and held the bell-button in one hand. S/He asked if the participant was ready and if s/he could give the first estimate. Then the RA looked down, pressed the button to indicate the movement part of his approach in the data, moved a 20 cm step forward to a subtly marked point on the catwalk, released the button to mark the stationary part of her/his approach and looked up at the participant. After the participant had given her/his estimation of the distance, this procedure was repeated until the RA was standing right in front of the participant at a distance of about 20 cm. This led to 16 stationary and 15 movement markers. After the last estimate the RA stepped off the catwalk to stop data-recording, while the participant remained on the platform. The RA stepped back on the catwalk at about 1.5 m and asked the participant to indicate a distance that s/he would find comfortable/would prefer for an interaction, by instructing the RA to move closer or further away. This preferred interpersonal distance (PID) was marked on the catwalk. Finally, the participant put on her/his shoes and was asked to fill in the remainder of the questionnaires, while the RA left the room. At the end of the experiment, participants were debriefed, compensated, and thanked. After the participant had left, the RA measured and noted the preferred IPS and wrote down the distance estimates by listening to the recordings.

### Data Preparation

The raw data from the 4 BB sensors was processed with MATLAB™ software (The Mathworks Inc., Natick, MA). From every participant the first 20 data-points (0.2 sec) were deleted. To determine a baseline from the empty BB, the following 20 data points were averaged. This mean was subtracted from each data-point with the participant on the BB to calculate a value that was solely determined by the weight of the participant and not by that of the BB. The last trial, in which the RA stopped at about 20 cm from the participant, was discarded from analyses, as this trial was terminated quicker than the previous ones to minimize the discomfort of the participants. For each of the remaining 14 distances (steps of the RA), we first calculated the mean Center of Pressure (COP)^2^2COP_AP = ((F1 + F2) – (F3 + F4) / (F1 + F2 + F3 + F4)) * (1000/2). COP_ML = ((F1 + F3) – (F2 + F4) / (F1 + F2 + F3 + F4)) * (1000/2). For sensor numbering, see Figure 1; 1000 represents the distance between the BB sensors in millimeters. in the Anterior-Posterior (forward-backward; COP_AP-Mean_) direction and in the Medio-Lateral (left-right; COP_ML-Mean_) direction. COP_AP-Mean_ was used to determine participants mean position/leaning towards the RA or backwards/away from the RA (i.e., “avoidance” per distance and per stance), COP_AP_ and COP_ML_ were both used to determine the general radius of body sway an indicator of the variability in body sway per distance and RA stance^3^3*R* = √(COP_x^2 + COP_y^2). We selected the radius because it provides a complete indication of changes in body sway (including diagonal movements), while the *SD* of, e.g, the COP_AP only indicates the radius of body sway in AP direction, deprived of the ML component.. A reduction in posture mobility is typically seen as index of “freezing” responses (Azevedo et al., 2005; Hagenaars et al., 2012; Roelofs et al., 2010a).

### Statistical Analyses

To investigate whether participants respond with avoidance and/or freeze behaviors when being approached and how these behaviors relate to degrees of social anxiety, separate Repeated Measures MANCOVAs were conducted: One for avoidance in AP direction with COP_AP-Mean_ direction as dependent variable, and one for magnitude of movement with movement radius as dependent variable. Distance in steps (14 à 20 cm) and the RA’s stance (move vs still) were independent within-subject variables. The total scores of LSAS and CES-D, participants’ PID, as well as their ratings of the RAs' friendliness, sympathy, attractiveness, and body scent were added as covariates. Whenever the assumptions of univariate testing were violated in any of the analyses, more conservative tests with corrections of degrees of freedom were used (i.e., Huynh-Feldt).

## Results

### Participants and Research Assistants

First, randomization of participants to RAs had been successful: There was no difference in gender ratio allocated to the female (28 female:14 male) or male (31 female:14 male) RA, χ^2^(1, *N* = 87) = 0.05, *p* = .83. In addition, a One-Way-ANOVA was used to test whether age, questionnaire-scores, and state measures differed between female and male participants. There were no significant differences on any of these measures between the genders, all *F*’s < 2.0, all *p*’s > .16 (Table 1). By means of a MANOVA it was explored whether female and male participants evaluated the RA differently, regarding friendliness, sympathy, attractiveness, or body scent. Again, no significant differences emerged, all *F*’s < 2.18, all *p*’s > .08. Finally, as would be expected, Pearson’s correlations revealed that degree of social anxiety (LSAS_min_ = 5, LSAS_max_ = 93) correlated positively with self-reported state anxiety at the beginning of the test session, *r*(87) = .28, *p* = .01, with tension/arousal, *r*(87) = .26, *p* = .02, and with number of depressive symptoms, *r*(87) = .58, *p* < .001.

**Table 1:** Mean (M), Standard Deviations (SD), of Age, Questionnaires, State Measures and Ratings of the Research Assistants per Participant Gender

	♀	♂	♀	♂	♀	♂	♀	♂	♀	♂
*n*	59	28	59	28	59	28	59	28	59	28
	Age		LSAS_Total_		CES-D_Total_		VAS_Anx._		VAS_Arousal_	
*M*	21.64	21.96	37.49	34.93	12.88	11.71	12.03	8.07	20.07	23.57
*SD*	3.41	2.63	17.90	13.22	10.03	8.02	16.35	7.88	17.85	21.41
	**VAS_Avoid_**		**RA_Friend._**		**RA_Symp._**		**RA_Attract._**		**RA_Odor_**	
*M*	11.08	8.57	73.69	74.36	68.27	69.14	37.53	42.64	58.10	54.71
*SD*	16.10	10.43	21.55	21.08	23.82	21.74	22.49	25.06	19.51	20.66

*Note.* LSAS = Liebowitz Social Anxiety Scale; CES-D = Center of Emotion Scale-Depression; VAS_Anx._ = Visual Analogue Scale (VAS) for State Anxiety; VAS_Arousal_ = VAS for State Arousal; VAS_Avoid_ = VAS for State Avoidance; RA_Friend._ = Reseach Assistant (RA) rating of friendliness; RA_Symp_ = RA rating of sympathy; RA_Attract._ = RA rating of attractiveness; RA_Odor_ = RA rating of body odor.

Four participants had to be excluded from further analyses because of erroneous BB data recordings, resulting in 83 participants for testing the main research questions.

### Interpersonal Distance, Social Anxiety and Avoidance (Leaning Backwards)

The only significant result concerned the three-way interaction of distance × RA stance × social anxiety on COP_AP-Mean_, *F*(12.04, 902.67) = 1.82, *p* = .041, η^2^ = .024. The remaining main effects and two-way interactions were not significant, all *F’*s < 2.52, all *p*’s > .11.

**Figure 2:**
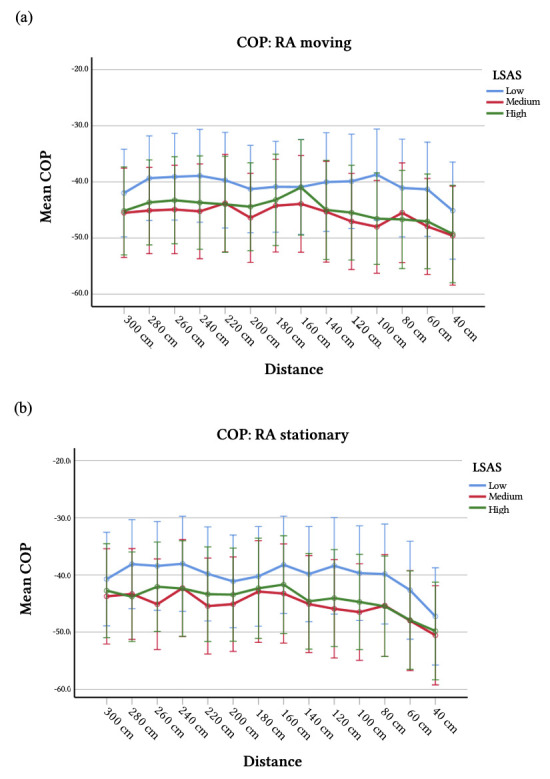
Mean Center of Pressure per Distance *Note*. Mean center of pressure in Anterior-Posterior direction (COP_AP-Mean_) and standard errors per distance to participant and participants’ degree of social anxiety as measured with Liebowitz Social Anxiety Scale (LSAS) for (a) Research assistant (RA) moving and (b) RA standing still. A more negative score reflects more leaning backwards/away from the RA.

To explore the three-way interaction in more detail, identical analyses were conducted but for the ‘moving’ and the ‘still’ stance separately. The critical interactions of distance × social anxiety were not significant *F’*s < 1.26, *p*’s > .26. The parameter estimates reveal that numerous distances of moving RAs are tendentiously related to social anxiety, while only two are for stationary RAs (Table 2). This difference between moving and standing still *may* have caused the observed three-way interaction but the conclusion must be that, neither social anxiety, distance to the participant nor the stance of the RA had any effect on the COP_AP-Mean_ (Figure 2: for *visualization* purposes participants were subdivided in three groups based on their LSAS scores: the 33.3% scoring lowest (score ≤ 29), 33.3% scoring highest (score ≥ 39), and those scoring in-between).^4^4Explorative analyses with these group divisions as between-subject factor revealed comparable results. In addition, based on suggestions of an anonymous reviewer, we conducted the originally planned analyses but added participant gender as between-subject factor and omitted preferred distance, RA friendliness, -sympathy, -attractiveness and -body odor as covariates. Again, the results did not change considerably.

**Table 2:** Parameter Estimates of Leaning Behavior per Distance and RA Stance Correlated With Social Anxiety Scores

Stance and Distance	*B*	*SE*	*t*	*p*	95% CI		η^2^
					*LL*	*UL*	
- Still -							
300 cm	-.262	.177	-1.478	.144	-.615	.091	.028
280 cm	-.304	.170	-1.791	.077^†^	-.643	.034	.041
260 cm	-.230	.170	-1.351	.181	-.570	.109	.024
240 cm	-.294	.180	-1.635	.106	-.652	.064	.034
220 cm	-.316	.177	-1.785	.078^†^	-.670	.037	.041
200 cm	-.226	.176	-1.282	.204	-.577	.125	.021
180 cm	-.282	.190	-1.489	.141	-.660	.095	.029
160 cm	-.265	.185	-1.432	.156	-.635	.104	.027
140 cm	-.288	.181	-1.591	.116	-.648	.073	.033
120 cm	-.282	.184	-1.529	.130	-.650	.085	.030
100 cm	-.278	.180	-1.540	.128	-.637	.081	.031
80 cm	-.296	.190	-1.562	.122	-.674	.082	.032
60 cm	-.235	.187	-1.259	.212	-.608	.137	.021
40 cm	-.144	.186	-.770	.444	-.515	.228	.008
- Moving -							
300 cm	-.271	.168	-1.607	.112	-.606	.065	.033
280 cm	-.188	.166	-1.130	.262	-.519	.143	.017
260 cm	-.305	.168	-1.812	.074^†^	-.639	.030	.042
240 cm	-.324	.180	-1.804	.075^†^	-.682	.034	.042
220 cm	-.332	.183	-1.810	.074^†^	-.697	.033	.042
200 cm	-.289	.169	-1.709	.092**^†^**	-.626	.048	.037
180 cm	-.239	.177	-1.351	.181	-.590	.113	.024
160 cm	-.101	.187	-.539	.592	-.473	.272	.004
140 cm	-.339	.191	-1.774	.080^†^	-.719	.042	.040
120 cm	-.311	.183	-1.699	.093^†^	-.675	.053	.037
100 cm	-.342	.175	-1.948	.055^†^	-.691	.008	.048
80 cm	-.339	.189	-1.796	.077^†^	-.714	.037	.041
60 cm	-.250	.184	-1.361	.177	-.616	.116	.024
40 cm	-.229	.189	-1.212	.229	-.606	.148	.019

**^†^***p* < .1. *p < .05.

### Interpersonal Distance, Social Anxiety and Body Sway (Freezing)

There was a non-significant trend of social anxiety, *F*(1, 75) = 3.57, *p* = .063, η^2^ = .045, indicating that increases in social anxiety were associated with more participant movement in general. The interaction of distance × social anxiety was significant, *F*(8.19, 614.34) = 2.33, *p* = .017, η^2^ = .03. This indicates that, with increasing levels of social anxiety, participants’ movability was increased as well, but only for some of the distances. All remaining main effects, two-way and three-way interactions were not significant, all *F*’s < 1.26, *p*’s > .10.^5^5Analyses conducted with the standard deviations of the COP_AP_ yielded similar results.

**Figure 3:**
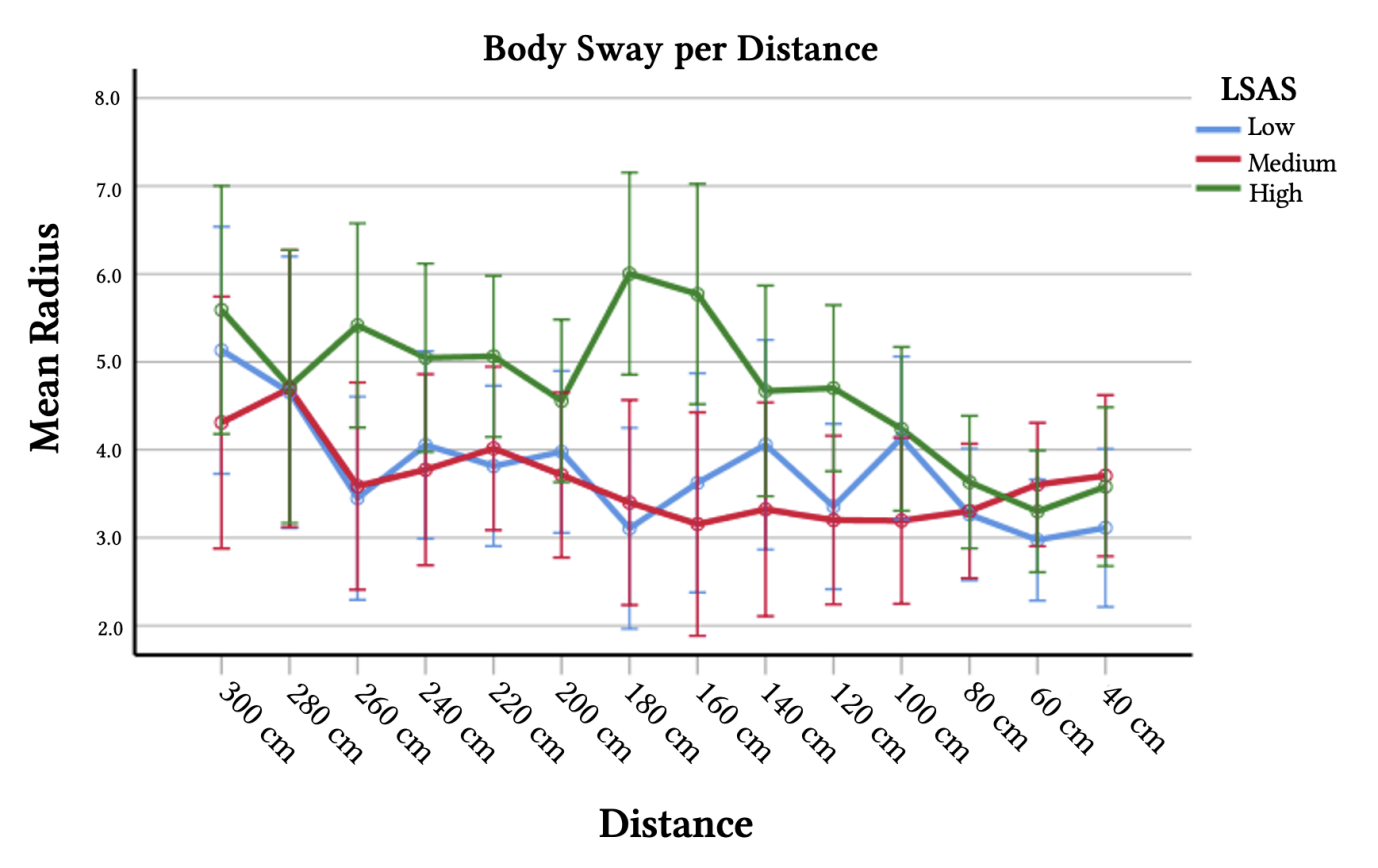
Mean Body Sway per Distance *Note.* Mean radius (Body Sway) per distance to participant and participants’ degree of social anxiety as measured with Liebowitz Social Anxiety Scale (LSAS). A higher score reflects more/larger movements in body posture irrespective of moving/stationary stance.

The parameter estimates revealed (marginal) significant effects of social anxiety at different distances (Table 3). In sum, it seems that SAs tend to move about more, when being approached by an unknown other. This movability seems to peek at about 180 cm and 160 cm distance between RA and participant (for visualization see Figure 3).

**Table 3:** Parameter Estimates of Body Sway per Distance and RA Stance Correlated With Social Anxiety Score

Stance and Distance	*B*	*SE*	*t*	*p*	95% CI		η^2^
					*LL*	*UL*	
- Still -							
300 cm	.009	.041	.215	.831	-.073	.090	.001
280 cm	.011	.051	.214	.831	-.092	.114	.001
260 cm	.078	.037	2.074	.042*	.003	.152	.054
240 cm	.010	.036	.285	.776	-.062	.083	.001
220 cm	.022	.026	.821	.414	-.031	.074	.009
200 cm	.008	.029	.260	.795	-.051	.066	.001
180 cm	.127	.039	3.244	.002*	.049	.206	.123
160 cm	.072	.028	2.530	.014*	.015	.129	.079
140 cm	.051	.039	1.324	.189	-.026	.128	.023
120 cm	.060	.025	2.401	.019*	.010	.110	.071
100 cm	-.019	.021	-.904	.369	-.061	.023	.011
80 cm	.030	.021	1.390	.169	-.013	.072	.025
60 cm	-.006	.017	-.351	.727	-.039	.028	.002
40 cm	.026	.027	.980	.330	-.027	.080	.013
- Moving -							
300 cm	-.004	.026	-.149	.882	-.056	.049	.000
280 cm	.024	.028	.853	.396	-.032	.079	.010
260 cm	.049	.023	2.138	.036*	.003	.095	.057
240 cm	.055	.023	2.368	.020*	.009	.102	.070
220 cm	.051	.027	1.938	.056^†^	-.001	.104	.048
200 cm	.024	.019	1.300	.198	-.013	.062	.022
180 cm	.019	.025	.784	.436	-.030	.069	.008
160 cm	.081	.039	2.107	.038*	.004	.158	.056
140 cm	.039	.019	2.011	.048*	.000	.078	.051
120 cm	.043	.022	1.991	.050^†^	-.000	.086	.050
100 cm	-.007	.029	-.229	.819	-.065	.051	.001
80 cm	.001	.018	.030	.976	-.035	.036	.000
60 cm	.012	.018	.689	.493	-.024	.049	.006
40 cm	-.017	.018	-.927	.357	-.053	.019	.011

**^†^***p* < .1. *p < .05.

### Preferred Interpersonal Distance and Social Anxiety

To explore the relationship between social anxiety and the preferred interpersonal distance (PID) when interacting with a stranger, Pearson’s correlations were calculated, two-tailed. Participants’ LSAS scores were not related to PID, *r*(87) = -.02, *p* = .83 (*M* = 73.37 cm, *SD* = 16.45; IPS_min_ = 45, IPS_max_ = 157).

### Explorative Analyses

To explore effects that are not (directly) related to the current research question, several additional analyses were performed.

#### Correlations

PID was negatively correlated with how attractive the RA was found, *r*(87) = -.26, *p* = .02: The more attractive the RA was evaluated, the closer participants positioned the RA to indicate their comfortable interpersonal distance. With regard to body sway it was found that mobility and evaluations of the RA’s body scent were positively correlated, *r*(87) = .24, *p* = .03: The more pleasant the RA’s body scent was, the more the participants moved about. It was also found that state arousal and mobility were negatively correlated, *r*(87) = -.22, *p* = .049: The more participants felt aroused and tense at the beginning of the test session, the less they moved about during the task.

#### Gender

To explore possible influences of participant gender, we also added this variable to the above-mentioned Repeated Measures MANCOVAs. This, however, had no influence on any of the statistical effects that were described above.

#### Distance Estimates

Finally, we explored whether the distance estimates that participants gave per step of the RA, were influenced by any of the relevant factors mentioned above. As would be expected, there was a main effect of distance *F*(1.87, 147.7) = 5.23, *p* = .008, η^2^ = .062, indicating that participants’ distance estimates declined with each step that the RA moved closer. No other relevant effect was found.

## Discussion

We investigated if decreasing interpersonal distance was related to subtle avoidance (backward leaning) and increased freezing-like behavior (reduced body sway), as well as the role of social anxiety here in. To test this, we used a stabilometric platform to measure objective movements accurately and applied it to a dynamic stranger-approach paradigm in a proof-of-principle study.

Against expectations, the distance between the approaching RA and the participant had no general effect on avoidant body posture or freezing-like behavior. Typically, one would have expected that an approaching, unfamiliar person would make participants retreat, at least when their personal space was breached. This was indeed partially demonstrated by Wieser et al. (2010). In a VR setting, participants made backward head movements and avoided eye gaze of approaching male digital agents at an uncomfortable 50 cm distance. However, they used fixed distances where the agents stopped (0.5 and 1.5 m) instead of dynamic distance changes as used in our study and in our study gaze avoidance was undermined by instructing participant to look the RA in the eyes. Yet, our study could have shown similar results as backward head movements should be readily reflected in our sensitive measure of body posture.

Unexpectedly, social anxiety did *not* have any influence on avoidant body posture when the interpersonal distance decreased. This is not in line with the results of Wieser et al. (2010) and Rinck et al. (2010). The embodied cognition theory suggest that automatic evaluative/emotional responses towards environmental stimuli are readily reflected in associated impulsive bodily responding (and vice versa: van Dantzig et al., 2009). Thus, higher degrees of social anxiety should have increased the salience of the experimental situation as social and potentially threatening, which should have been reflected in automatic bodily avoidance. Numerous studies using prototypical approach-avoidance tasks based on joystick-movements towards social stimuli, found such automatic avoidance behavior towards angry but not neutral faces in highly socially anxious individuals (e.g., Heuer et al., 2007; Lange et al., 2008; Roelofs et al., 2010b). It is possible that particularly angry faces as representation of prototypical threat evoke threat-related avoidance responses in SAs while approaching, neutral looking RAs might be merely unpleasant but inapt to evoke threat responding (Lange et al., 2014) as observed in our study.

Body sway, on the other hand, *was* influenced by participants’ degree of social anxiety interacting with the distance of the RA. Interestingly, the pattern was opposite to what was expected: Participants with high levels of social anxiety showed increased bodily movement at 260cm, and between 200cm and 100cm distance, with a peak at 180cm. These results seem to contradict numerous previous studies that found reduced movement in response to threat in unselected samples (e.g., Azevedo et al., 2005; Hagenaars et al., 2014b; Roelofs et al., 2010a). Studies with participants showing characteristics related to *social* anxiety, however, reported more contradictory results. For example, while insecure attachment was associated with *increased* freezing-like responding (Niermann et al., 2015), internalizing symptoms were not (Niermann et al., 2017). Two studies found no effects of stimulus valence but instead general reductions of body sway throughout the experiment (social anxiety disorder: Levitan et al., 2012; panic disorder: Lopes et al., 2009), and one study found reduced body sway for blocks with painful stimuli, but not modulated by social context (Karos et al., 2020). Importantly, there is also evidence for a *lack* of freezing behavior in psychopathology. Stoffels et al. (2017), for example, reported reduced heart rate (typically associated with freezing) in response to aversive pictures in healthy controls but not in patients with borderline personality disorder. In the same line, Fragkaki et al. (2017) discovered attenuated freezing responses in patients with posttraumatic stress disorder. Finally, Hagenaars et al. (2015) found that heart rate reductions (indicative of freezing) were absent when participants were prepared for the aversive stimuli but not when being unprepared. Warning participants in the current study about the (very intimate) approach of the RA might have prevented the evaluation of the situation as threatening and may have undermined typical threat related responding. This is also compatible with Fanselow’s (2022) idea that context and type of threat can influence the timing, type and degree of defensive responding. Taken together with Trower and Gilbert's (1989) evolutionary approach that social anxiety is not so much determined by imminent threat of death but more by survival related resources, freezing may be less likely in the approaching stranger context than other behavior that signals discomfort or unease.

Note that higher degrees of *state* anxiety at baseline, irrespective of social anxiety, *were* associated with decreased body sway/freezing, indicating that our measure was sensible enough to pick up freezing-like behavior when participants *are* stressed. This is in line with previous work from, e.g., Hagenaars et al. (2014b) or Roelofs et al. (2010a). It might be that particularly state assessments of subjective experiences of stress/fear are more directly linked to physiological and automatic behavioral responding. Our assessment of SA, however, is more based on anxiety traits that may not necessarily be related to the stress levels in this particular situation. True is, that, in general, state stress and trait SA are positively correlated, but the predictability of our specific procedure may have evoked an attitude often observed with high functioning SAs: knowing the job, getting it done, with nervous fidgeting and restless movements indicating discomfort (Heerey & Kring, 2007; Voncken et al., 2008, 2012) but no pronounced fear reaction. In line with this notion, research has indicated that SA is a heterogeneous condition based, under more, on distorted interpretations and evaluations of environmental cues and social situations (Clark, 2001; Hofmann, 2007; Rapee & Heimberg, 1997). As indicated above, Fanselow (2022) as well as Trower and Gilbert (1989) acknowledge that decreases in adaptive defense thresholds are highly related to subjective evaluations and interpretations of the context. It is therefore thinkable that high degrees of social anxiety are generally related to increased stress in a situation, but that it is primarily the degree of SA in these healthy participants that allows for evaluating the situation as unpleasant but not threatening which then could determine their subtle behavior accordingly. It is important to note that the social anxiety levels of this particular sample were unexpectedly higher than is typical of a student sample. If the range between a score of 30–60 on the LSAS marks mild to moderate degrees of social anxiety then about 54% of the participants fall in that range. About 10% score 60 or higher which might be indicative of a clinically relevant degree of social fears (Mennin et al., 2002; Rytwinski et al., 2009).

In sum, increases of body sway may be more indicative of general unease with or nervousness in a situation rather than reflecting the expectation of eminent threat. For our understanding of social anxiety, this could mean that SAs show subtle signs of discomfort in a social situation. Once noticed by interaction partners they may feel uncomfortable themselves and may start disliking the situation, thereby fulfilling the SAs ‘prophecy’ of being disliked by others (Asher et al., 2020; Tschacher et al., 2014).

Remarkably, we found differences in body sway between high and low socially anxious individuals when the RAs were at a moderate distance. That is, RA-distance related changes in body sway were in the form an inverted U-shape. Especially on the uncomfortably short distances, one would have expected anxiety related effects to be strongest. This may have been a floor effect: The unnaturally close interpersonal distances are likely to be perceived as highly unpleasant for everyone, irrespective of social fears (Bailey et al., 1972; Dosey & Meisels, 1969; Hayduk, 1983). But exaggerated threat interpretations and resulting anxiety responses in social anxiety become primarily apparent in ambiguous situations and not necessarily in situations that actually *are* highly uncomfortable or threatening. In addition, subtle signs of discomfort and unease, rather than obvious ones, would manifest in a distance range where normal social interactions take place and not at a far distance, when there is clearly no social threat.

Irrespective of these results, a few limitations need to be considered. As indicated above, the predictability of what participants could expect may have diminished the experience of threat and related behaviors. Luo et al. (2019) found indeed that task instructions can readily attenuate automatic responding to emotional stimuli. In addition, our participants were, of course, free to leave the situation whenever unbearable while freezing-like behavior may be more likely to occur without (knowledge of) escape options (compare: Clark, 1993; Sanderson et al., 1989). For example, Buss et al. (2004) found that children responded with more freezing to an approaching stranger when they were restrained in a high-chair instead of freely playing. Finally, while our setup was considerably more dynamic than, e.g., that of Wieser et al. (2010), approaching participants in steps rather than in a continuous motion in combination with distance judgements may still be suboptimal and decrease ecological validity. Future research should delineate specific responses to dynamic social interactions and distinguish social threat from social discomfort herein to provide the most ecologically validity when measuring subtle behavior. Although not consistently supported in SA research (e.g., Cheng et al., 2022), the addition of physiological measures to the current study setup may help to further the understanding of subtle behaviors in social interactions.

To conclude, with our new experimental setup, we found that high degrees of social anxiety were associated with deviations from subtle ‘default’ behaviors seen as normal in social interaction: SAs showed increased movement when their own personal space was intruded. Future research may explore whether such behavior elicits feelings of discomfort in others, and if so, whether that would lead to *true* negative evaluation of SAs. Treatments may target these subtle behaviors to break this vicious circle. If psychotherapy, “primarily tackles faulty cognitions while there is a considerable probability that the fear of negative evaluation is justified, chances of substantial and sustained recovery are undermined.” (Lange et al., 2014, p. 360).

## Data Availability

Research material and data will be available upon request.

## Reporting Guidelines

To the best of our knowledge we adhered to the JARS Quant guidelines of reporting quantitative research.

## Preregistration

In the time that the study was conducted, preregistrations and a priori power analyses were rather uncommon, and thus not done for the current study.
